# [Expression of Concern] *In vitro* and *in vivo* activities of an antitumor peptide HM-3: A special dose-efficacy relationship on an HCT-116 xenograft model in nude mice

**DOI:** 10.3892/or.2026.9191

**Published:** 2026-09-10

**Authors:** Sitelbanat Yassin, Jialiang Hu, Hanmei Xu, Ce Li, Sarra Setrerrahmane

Oncol Rep 36: 2951–2959, 2016; DOI: 10.3892/or.2016.5077

Following the publication of the above article and a corrigendum (doi: 10.3892/or.2020.7503) that was published to take account of duplications of data noted in Fig. 3 (panels ‘C’ and ‘D’) and Fig. 7 (also panels ‘C’ and ‘D’), it has come to light that the originally published version of Fig. 7 also contained a small overlapping section of data comparing Fig. 7A with 7D.

Upon receiving notification of this further issue associated with the presentation of data in Fig. 7, owing to the non-availability of the first author who performed these experiments and the inability to identify the original data for this figure, the authors proposed removing the data from Fig. 7A-D, and simply presenting Fig. 7E in a revised version of this figure in a subsequent corrigendum; however, the Editor of *Oncology Reports* requested that the authors repeat the experiments shown in Fig. 7. and submit a new and complete version of this figure for the corrigendum. The authors were agreeable to this course of action, although to date, the proposed revised version of this figure has not been received by the Editorial Office; therefore, we are now issuing an Expression of Concern statement to notify readers of the newly identified problems associated with the presentation of certain of the data in this paper.

